# P01 The increasing incidence of Gram-negative bacteraemias in the UK: confounding effects of the Sepsis 6 campaign

**DOI:** 10.1093/jacamr/dlac004

**Published:** 2022-02-16

**Authors:** Ioannis Baltas, Jane Democratis

**Affiliations:** Department of Infectious Diseases and Microbiology, Frimley Health NHS Foundation Trust, Berkshire, UK; Department of Infectious Diseases and Microbiology, Frimley Health NHS Foundation Trust, Berkshire, UK

## Abstract

**Introduction:**

The 2019–20 English Surveillance Programme for Antimicrobial Utilisation and Resistance Report (ESPAUR) stated that the incidence of bacteraemias in key bacterial species has increased 17% between 2015 and 2019, continuing the trend observed since mandatory national reporting of *Escherichia coli* bacteraemias was initiated in 2011. PHE estimates the incidence of infections by calculating the rate of bacteraemias per 100 000 population or per 100 000 bed days for acute Trusts. We hypothesized that the incidence of infections is also affected by the number of blood cultures performed, given that collection of blood cultures for critically unwell patients has been persistently promoted in the UK during the 2010s as part of the Sepsis 6 bundle.

**Methods:**

We compared the incidence of *E. coli* bacteraemias in an acute NHS Trust from financial year 2012–13 through to 2018–19, with and without adjusting for the number of blood cultures performed, using centrally reported Trust data.

**Results:**

During the study period, the total number of *E. coli* bacteraemias increased from 375 to 691 (84.3%, Table 1). At the same time, the total number of blood cultures performed increased from 18 956 to 32 283 (70.3%), while the total overnight bed occupancy increased from 377 029 to 453 191 (20.2%, Table 1). The bed occupancy adjusted incidence of *E. coli* bacteraemias increased from 99.5 E. bacteraemias per 100 000 occupied bed days in financial year 2012–13 to 152.5 bacteraemias per 100 000 occupied bed days in 2018–19 (53.2%, Table 2). The blood culture adjusted incidence of *E. coli* bacteraemias decreased from 52.5 *E. coli* bacteraemias per 100 000 bed days per 10 000 blood cultures in 2012–13 to 47.2 *E. coli* bacteraemias per 100 000 bed days per 10 000 blood cultures in 2018–19 (−10%, Table 2). Even when excluding the 2012–13 financial year, which looks like an outlier, the blood culture adjusted incidence of *E. coli* bacteraemias only increased by 10% between financial years 2013–14 and 2018–19 (Table 2). Correlation analysis showed a strong and statistically significant association between blood cultures performed and *E. coli* bacteraemias per 100 000 bed days (R = 0.971, R^2^ = 0.9428, *P<*0.001).

**Conclusions:**

The number of blood cultures performed is a potential confounder behind the recent increase in the incidence of *E. coli* bacteraemias. Regulators should consider adjusting for this factor when reporting incidence of these infections.

**Table 1.** Annual *E. coli* bacteraemias, blood culture and overnight bed occupancy totals of Frimley Health NHS Foundation Trusts for financial years 2012–13 until 2018–19

Financial year	Total *E. coli* bacteraemias	Blood cultures per annum	Overnight beds occupied per annum
2012–13	375	18 956	377 029
2013–14	360	20 513	408 867
2014–15	444	22 887	431 792
2015–16	500	24 510	448 130
2016–17	610	29 067	468 153
2017–18	701	32 537	464 409
2018–19	691	32 283	453 191

**Table 2.** Annual *E. coli* bacteraemia incidence when correcting or not correcting for blood culture numbers of Frimley Health NHS Foundation Trusts for financial years 2012–13 until 2018–19

Financial year	*E. coli* bacteraemias per 100 000 occupied bed days	*E. coli* bacteraemias per 100 000 occupied bed days per 10 000 blood cultures
2012–13	99.5	52.5
2013–14	88.0	42.9
2014–15	102.8	44.9
2015–16	111.6	45.5
2016–17	130.3	44.8
2017–18	150.9	46.4
2018–19	152.5	47.2

**Figure 1.**
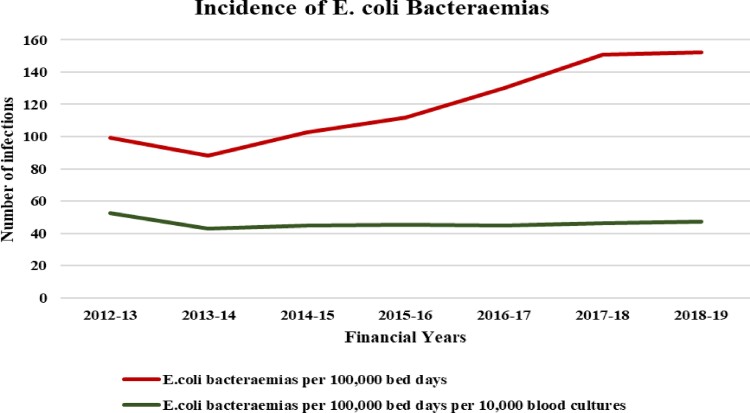
Annual *E. coli* bacteraemia incidence when correcting or not correcting for blood culture numbers of Frimley Health NHS Foundation Trusts for financial years 2012–13 until 2018–19.

